# The effectiveness of decompression as initial treatment for jaw cysts: A 10-year retrospective study

**DOI:** 10.4317/medoral.22526

**Published:** 2018-12-24

**Authors:** Saša Marin, Barbara Kirnbauer, Petra Rugani, Alexandra Mellacher, Michael Payer, Norbert Jakse

**Affiliations:** 1 Doctor of Dental Medicine, Oral surgery specialist, Department of Oral Surgery, Faculty of Medicine, University of Banja Luka; 2 Doctor of Dental Medicine, Division of Oral Surgery and Orthodontics, Department of Dental Medicine and Oral Health, Medical University of Graz; 3 Associate Professor, Division of Oral Surgery and Orthodontics, Department of Dental Medicine and Oral Health, Medical University of Graz; 4 Full Professor and Head, Division of Oral Surgery and Orthodontics, Department of Dental Medicine and Oral Health, Medical University of Graz

## Abstract

**Background:**

Decompression is an approved alternative to cystectomy in the treatment of jaw cysts. This study aimed to evaluate its effectiveness as an initial procedure, as well as factors with potential to influence outcome.

**Material and Methods:**

The frequency of decompression was analysed, whether completed in one session or followed by enucleation at the Division of Oral Surgery and Orthodontics, Department of Dental Medicine and Oral Health, Medical University of Graz, from 2005 to 2015. Further analysis focussed on factors potentially influencing outcome: cyst location, histopathology, means of preserving the cyst opening, cyst size, patient age.

**Results:**

In all, 53 patients with 55 jaw cysts (mean age of 35.1) were treated by initial decompression in the ten-year period. In the majority of cases, histopathological analysis revealed a follicular cyst (43.6%), followed by odontogenic keratocysts (23.7%), radicular cysts (21.8%), residual cysts (7.3%) and nasopalatine cysts (3.6%) Treatment was completed with a single decompression in 45.5% of the cases. Among those, 72.0% were follicular cysts and 8.0% odontogenic keratocysts. Subsequent enucleation was needed in 54.5% of all cases, with a majority in the keratocystic group (36.7%). Histological findings, means of keeping the cyst open, and patient age were found to influence the effectiveness of decompression.

**Conclusions:**

Decompression could be performed as a procedure completed in one session or combined with subsequent enucleation, mainly dependent on histopathological findings. Subsequent enucleation of odontogenic keratocysts is highly recommended.

** Key words:**Jaw cysts, decompression, enucleation, histopathology, obturator.

## Introduction

Cystic lesions occur more frequently in the upper and lower jaws than in other bones of the human body, mainly due to the presence of cells that are remnants of the embryonal neuroectoderm. One further explanation is that the embryonic teeth are located in the jaw bones. Triggers are either inflammatory stimuli or developmental disorders (1). Because they are usually slow growing and asymptomatic, cysts may grow very large, displacing and even damaging surrounding structures, with subsequent infection, root resorption, nerve injuries or bone fractures (2,3).

Treatments range from single decompression, marsupialization, enucleation and bone resection to a combination of these approaches (4,5). While there is no consensus on optimal treatment, complications and further morbidity are to be avoided, particularly with large cysts.

Decompression as initial procedure is a common conservative approach requiring preparation and preservation of a cyst opening. The aim is to decrease intracystic pressure by constant drainage, so allowing new centripetal bone growth from the bony cyst walls (6). The cyst opening can be preserved with simple iodoform gauze packing, a custom-made obturator, bracket and chain on involved impacted teeth, and drains (7,8).

The main advantages of decompression are that it spares tissue, minimizes the likelihood of damage to adjacent structures, and avoids the cost of hospitalization (9,10). Complications have been reported more frequently when enucleation was performed as a single procedure for extensive jaw cysts. According to the literature, the prevalence of permanent sensory disturbance ranges from 2.0-18.0%, of transient hypoesthesia from 8.0-35.0%, and of incomplete ossification from 12.0-40.0% (11-15).

Disadvantages of decompression include the duration of treatment, discomfort, and reliance on patient compliance. Further, remnants of the epithelial lining can lead to cyst recurrence requiring further surgical treatment (16,17).

Some authors have suggested subsequent enucleation for aggressive cysts with a high relapse rate, and when the outcome of decompression is unsatisfactory (18,19).

This retrospective study aimed to evaluate the effectiveness of decompression for treatment of jaw cysts with consideration of possible outcome-influencing factors including patient age, cyst location and size, histopathology, and means of preserving the cyst opening.

## Material and Methods

After approval of the study by the local ethics committee, data were collected and analysed from patients who had undergone decompression at the Division of Oral Surgery and Orthodontics, Department of Dental Medicine and Oral Health, Medical University of Graz, from 2005 to 2015.

The inclusion criteria for the study were cyst in the upper or lower jaw treated with decompression and complete medical records.

The exclusion criteria were cyst in the upper or lower jaw treated initially with enucleation or resection, soft tissue cysts, and incomplete medical records.

The following data were collected and analysed: frequency of decompression and decompression followed by enucleation, patient’s age and gender, location and size of the cyst, histopathological findings, means of preserving the cyst opening.

Histopathology reports were obtained from the Institute of Pathology of the Medical University of Graz.

After surgical decompression, the cyst was kept open with iodoform gauze for the first few postoperative days. Thereafter, besides continued gauze packing, obturators, brackets with chains, and drains were used. Patients were advised to follow all postsurgical instructions scrupulously, rinsing the cyst opening twice a day with 0.9% NaCl solution using a syringe, and cleaning dental devices mechanically with a toothbrush or swabs. For the first 2 days, postoperative care included cryotherapy with cold packs and for pain management, dexibuprofen 200 mg for children and 400 mg for adults (Seractil® 200 or 400mg, Gebro Pharma, Austria) 3 times a day. Routine follow-up included clinical and radiological studies at least every three months. Additional appointments were arranged depending on individual needs and compliance. Digital panoramic x-rays were taken with the Orthophos XG plus DS (Sirona Dental Systems GmbH, Bensheim, Germany) with 60–70kVp and 14-17 mA.

**Figure 1:**
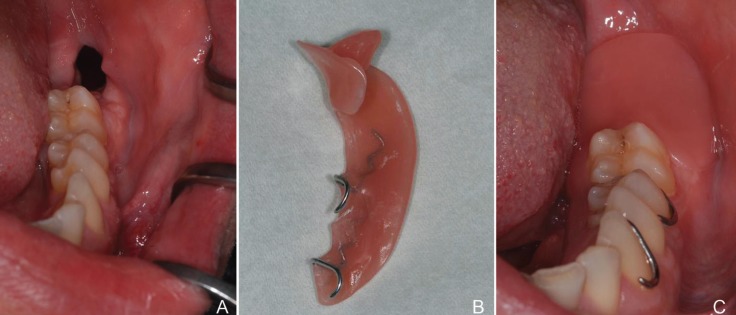
(A) the cyst opening 7 days after decompression; (B) customized obturator; (C) applied customized obturator.

**Figure 2:**
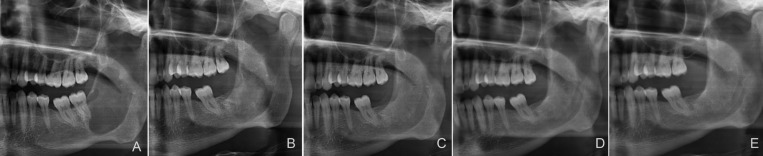
(A) panoramic radiograph of odontogenic keratocyst before decompression; (B) panoramic radiograph 1 year after decompression and before subsequent enucleation; (C) panoramic radiograph 1 year after subsequent enucleation; (D) panoramic radiograph obtained 2 years after subsequent enucleation; (E) panoramic radiograph 5 years after subsequent enucleation.

Those patients with insufficient cyst shrinkage after decompression later underwent enucleation (Figs. 1,2).

-Statistical analysis

Data were presented with descriptive statistics. The statistical analyses were performed with SPSS software (IBM SPSS statistics 24.0, IBM Corporation, New York, United States) at a 5% significance level. The chi-square test and Student’s t-test were applied to quantitative and continuous variables.

## Results

There were 53 patients (55 cysts) in the ten-year study period with a mean age of 35.1 years; 39 were male (73.6%) and 14 female (26.4%) ( Table 1). Cysts were more common in the mandible (38 cysts) than in the maxilla (17 cysts). Mandibular cysts were most often found in the posterior region (molar-retromolar (34.2%) followed by retromolar (23.7%)), while maxillary cysts were most common in the frontal region (35.2%) ( Table 2). Follicular cysts were most frequent (43.6%), followed by odontogenic keratocysts (23.7%), radicular cysts (21.8%), residual cysts (7.3%) and nasopalatine cyst (3.6%). Most commonly, an obturator was used to keep the cyst open (54.5%), followed by bracket with chain (25.5%), iodoform gauze packing (14.5%), and drain (5.5%) ( Table 3).

**Table 1:**
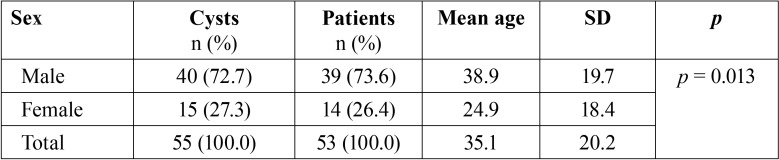
Descriptive patient data.

**Table 2:**
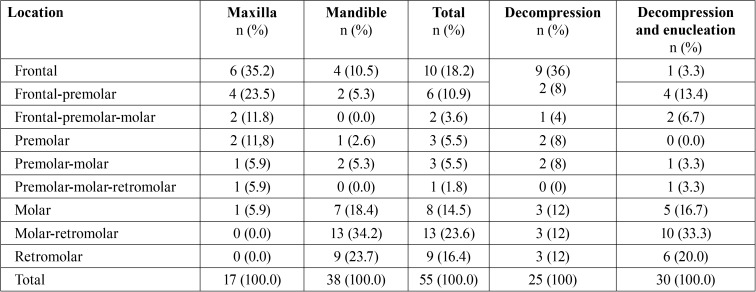
Distribution of the cysts and surgical procedures by jaw location.

**Table 3:**
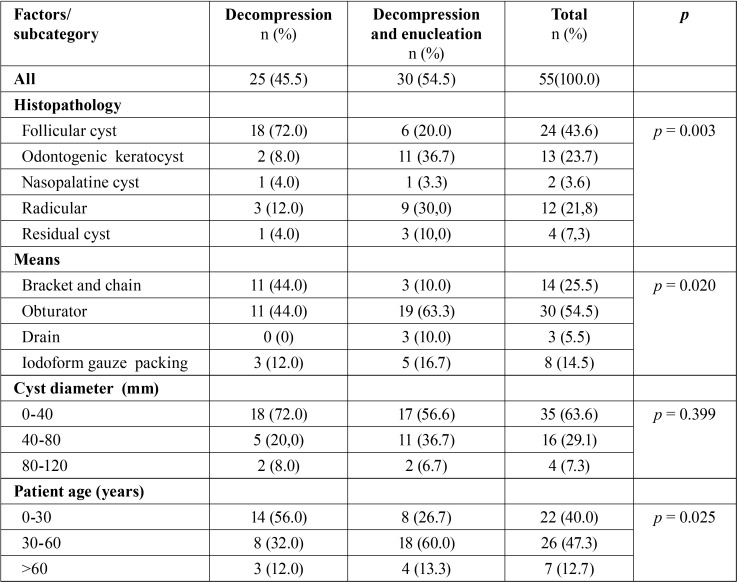
Relation between different factors and types of the surgical procedures.

A single decompression completed treatment in 45.5% of cases, mostly in the frontal region of the jaws and in patients under 30 years (56.0%). Among these patients, follicular cysts were most frequent (72.2%) and the most commonly used devices were brackets with chains (44.0%) ( Table 3).

Subsequent enucleation was needed in 54.5% of the cases, mostly in the posterior region. Patients were usually 30-60 years old (60%) and odontogenic keratocysts (36.7%) were most common. An obturator was often used after decompression followed by enucleation (63.3%) ( Table 3).

The effectiveness of decompression was found to correlate with histopathology, the means of keeping the cyst open, and patients’ age (*p*=0.003, *p*=0.020 and *p*=0,025). More detailed information is presented in  Table 3.

The cyst’s diameter was not found to have an influence on the effectiveness of the procedure (*p*=0.399) ( Table 3).

## Discussion

This retrospective study focused on the evaluation of the effectiveness of decompression for jaw cyst treatment over a ten-year period and the influence of different factors thereon. The main limitations of this study were that it was retrospective and that medical data were not always complete.

The study included 53 patients with a mean age of 35.1 years and 55 cystic lesions treated initially with decompression. In accordance with the literature, the most frequent cystic lesions occurred in anterior maxilla in male patients (20-22), though there were more cysts overall in the mandible than the maxilla. The reason for the higher frequency of the cysts in lower jaw could be the use of enucleation as the initial treatment for cystic lesions in maxilla, while this study focused on jaw cysts initially treated with decompression.

Similarly, histopathologically, follicular cysts and odontogenic keratocysts were most frequent (43.6% and 23.7%, respectively). The literature indicates that radicular cysts are the most frequent cysts in the jaws (23). Radicular cysts are smaller and are initially treated with enucleation. Only large radicular cysts are treated with decompression when enucleation could damage surrounding structures, or in the case of geriatric and high-risk patients. The frequencies of residual cysts and nasopalatine cysts of 7.3% and 3.6%, respectively, are in line with the literature averages of 4.2-13.7% and 2.2-4.0% (20,22). Histopathological findings showed that the cyst type influences the surgical approach (*p*=0.003), with decompression followed by enucleation applied mostly for odontogenic keratocysts. As a single procedure, decompression was most frequently used for follicular cysts (72%), but only for 8% of odontogenic keratocysts. Some authors have advocated decompression for odontogenic keratocysts (24), although surgeons often prefer decompression followed by enucleation for these aggressive cysts that are highly prone to recurrence (18).

Various means of preserving the cyst opening have been described in the literature (19,24), but there is little information on their comparative effectiveness. In this study, a statistically significant difference was found in the use of means of preserving the cyst opening and the frequency of decompression or decompression followed by enucleation (*p*=0.020). Decompression showed more success when brackets and chains were used rather than other devices. The reason could that brackets with chains are mostly used for follicular cysts. Although obturators can be custom made, it may not be easy to create a precise obturator due to the position of the opening in the mouth, tissue remodelling and imprecise impressions of the inner lumen. Iodoform gauze packing and drain tubes might be used less often due to the surgeon’s preference and the need for extensive after-care. Some patients have difficulty keeping appointments to have their iodoform gauze changed, while others may be over-challenged with keeping a drain clean (11).

The management of various sizes of the cysts continues to be discussed. Jeong *et al.* concluded that the initial size of the cyst influences the outcome of decompression (25), while Anavi *et al.* did not find any influence of the initial cyst’s size on the outcome of decompression, which is in accordance with our results (*p*=0.399). However, decompression followed by enucleation was used more often when the cyst’s diameter exceeded 40 mm. Jeong *et al.* made three-dimensional cyst measurements while Anavi *et al.* made cyst measurements on panoramic records (11,25). A shortcoming of this study could be measuring the largest cyst diameter on panoramic records and not taking into account the buccal-lingual dimensions of the cystic lesions.

The relationship between patient age and the reduction rate of the cyst’s size after treatment is still unclear. Some studies have found patient age to be an important factor in the cyst’s healing process (26,27), while others have failed to find a correlation (28,29). In this study, patient age seemed to have an effect on the surgical treatment chosen (*p*=0.025). Decompression was more successful in patients under 30 years of age than in older patients, which could be explained by the higher occurrence of follicular cysts in younger patients. Follicular cysts are not as aggressive as odontogenic keratocysts, which is why the process is likely to succeed when decompression is the chosen treatment.

## Conclusions

Decompression is mainly performed to avoid morbidity. It can be performed as a single complete procedure or combined with subsequent enucleation, mainly depending on histopathological findings. Enucleation after decompression is highly recommended for odontogenic keratocysts.
